# Gray Matter Atrophy to Explain Subclinical Oculomotor Deficit in Multiple Sclerosis

**DOI:** 10.3389/fneur.2019.00589

**Published:** 2019-06-04

**Authors:** Bálint Kincses, Benjámin J. Hérák, Nikoletta Szabó, Bence Bozsik, Péter Faragó, András Király, Dániel Veréb, Eszter Tóth, Krisztián Kocsis, Krisztina Bencsik, László Vécsei, Zsigmond Tamás Kincses

**Affiliations:** ^1^Department of Neurology, Albert Szent-Györgyi Clinical Center, University of Szeged, Szeged, Hungary; ^2^MTA-SZTE Neuroscience Research Group, Szeged, Hungary; ^3^Department of Radiology, Albert Szent-Györgyi Clinical Center, University of Szeged, Szeged, Hungary

**Keywords:** multiple sclerosis, prosaccade, anti-saccade, atrophy, black hole

## Abstract

Eye movement deficits are frequently noted in multiple sclerosis during bedside clinical examination, but subtle dysfunction may remain undetected and might only be identified with advanced approaches. While classical neurology provides insight into the complex functional anatomy of oculomotor functions, little is known about the structural background of this dysfunction in MS. Thirty four clinically stable, treated relapsing-remitting MS patients with mild disability and 34 healthy controls were included in our study. Group difference and correlation with clinical parameters were analyzed in case of the latency, peak-velocity, gain, dysconjugacy index, and performance during a saccade and anti-saccade task. High-resolution T1 weighted, T2 FLAIR, and double inversion recovery images were acquired on 3T to evaluate the correlation between behavioral and MRI parameters, such as T2 lesion and T1 black-hole burden, global brain, gray, and white matter atrophy. VBM style analysis was used to identify the focal gray matter atrophy responsible for oculomotor dysfunction. Significantly increased latency in the prosaccade task and significantly worse performance in the anti-saccade task were found in MS patients. The detailed examination of conjugated eye movements revealed five subclinical internuclear ophthalmoparesis cases. The peak velocity and latency of the anti-saccade movement correlated with the number of black holes, but none of the eye movement parameters were associated with the T2 lesion burden or location. Global gray matter volume correlated with saccade and anti-saccade latency, whereas white matter and total brain volume did not. Local gray matter atrophy in the left inferio-parietal lobule and temporo-occipital junction correlated with anti-saccade peak velocity. Our results show that neurodegeneration-like features of the MRI (black-hole, gray matter atrophy) are the best predictors of eye movement deficit in MS. Concurring with the clinico-radiological paradox, T2 lesion burden cannot explain the behavioral results. Importantly, anti-saccade peak velocity correlates with gray matter atrophy in the left parietal regions, which are frequently implicated in attention tasks.

## Introduction

Multiple sclerosis (MS) is a devastating disease that mostly affects young adults. Among many other symptoms, oculomotor deficit is common in MS, reported to occur in 57–70% of all patients (1, 2). Its significance lies in the observation that the presence of eye movement abnormality is associated with greater disability and greater disability progression (3). Bedside oculo-motor examination by an experienced specialist could reveal major oculomotor deficits, but subtle alterations might remain undetected. Eye tracker devices are suitable for objective and quantitative measurements of eye movements and are more sensitive in detecting subclinical abnormalities (1).

The aim of the eye movements is to keep the object of interest on the fovea. These voluntary and reflexive movements are the rapid jerky saccades, the smooth pursuit and vergence movements. These intricate ocular movements are accomplished by six extraocular muscles, the movement of which is coordinated by a complex network of cortical and subcortical neuronal elements.

The main purpose of the rapid voluntary conjugate eye movements known as saccades, is to bring the new object of interest onto the foveae. The cranial nerve nuclei of the oculomotor muscles could be found in the brainstem. In addition, other elements of the premotor circuits of saccades are in the brainstem such as the paramedian pontine reticular formation, nucleus of the medial longitudinal fasciculus and nucleus raphe interpositius. All of these regions receive afferents from the superior colliculus. Moreover, the behaviorally important stimuli are processed in various cortical networks and together with fronto-parietal attention networks have crucial role in guiding eye movements during saccades (4). Eventually the cortical signals for eye movements are generated in the frontal eye filed (FEF) in close interaction with other centers such as supplementary and pre-supplementary eye fields (5), the dorsolateral prefrontal cortex and parietal cortex. From the cortical centers the information is conveyed via the superior colliculus to the nuclei of the oculomotor nerves directly and indirectly as well (6–8). Over the course of information flow various subcortical, brainstem and cerebellar centers are modulating the process.

Damage to certain parts of this network causes clinically abnormal eye movement (9, 10) some of those easily detectable by bedside examination (11). However, the structural background of subtle eye movement deficits in MS is not well-understood. Damage of the perceptual systems, the cognitive networks such as attention and the eye movement centers cause various alterations of eye movements.

The aim of our study was to investigate the subclinical oculomotor deficit of MS patients in prosaccade and anti-saccade tasks. The higher order structural background of such abnormal eye movements was investigated by correlating the behavioral measures with lesion location and gray matter atrophy.

## Materials and Methods

### Subjects

Thirty nine relapsing remitting MS patients were enrolled in our study. Inclusion criteria for patients were: relapsing remitting MS on disease modifying treatment, EDSS score < 6, no relapse in the preceding 3 months, no other major neurological, psychiatric or ophthalmological disease (for clinical and demographic data see Table 1).

**Table 1:** Demographic data of the subjects.

**Group**	**# of subjects**	**Females**	**Age (years)**	**EDSS**	**Disease duration (months)**	**Treatment regimen**
MS	39	25	39.1(±9.5)	1.4(±1.4)	103(±72.8)	DF-15% Te-28% IFNb-13% GA-18% F-26%
HC	34	23	31(±10.9)	–	–	–

*DF, dimethyl fumarate; Te, teriflunodamide; IFNb, interferon beta 1a; GA, glatiramer acetate; F, fingolimid*.

We also recruited 34 healthy controls (HC), who had no major neurological, psychiatric, or ophthalmological disease.

This study was carried out in accordance with the recommendations of the Medical Research Council National Scientific and Ethical Committee (ETT TUKEB) with written informed consent from all subjects. All subjects gave written informed consent in accordance with the Declaration of Helsinki. The protocol was approved by the National Institute of Pharmacy and Nutrition (000002/2016/OTIG).

### Visuo-Motor Task

The subjects completed a prosaccade and an anti-saccade task. The investigation took place in a well-lit room. The subjects sat 60 cm away from the screen. The visual stimuli and the task paradigm were written using the Tobii MATLAB binding^1^ and the Psychophysics Toolbox Version 3.0.12^2^, under MatLab 8.3.0.532 (2014a, MathWorks, Inc.). Eye movement recording was carried out with a Tobii TX300 eyetracker. Before the task, a 5 points calibration was carried out. The prosaccade task was the following: A black cross appeared in the center of a gray screen, which disappeared after a random interval of 1.2–2 s and appeared instantaneously in the left or right side of the screen, 9.2 or 18.4°From the center. Each condition (4 in all: left-far, left-close, right-far, right-close) was repeated 20 times in a pseudorandom order. Subjects had to move their gaze to the new location of the target instantly and accurately. Halfway during the task, there was a break to prevent subjects from fatigue and/or tearing. In the anti-saccade task, the layout was the same, but the subjects had to move their gaze contralateral to the position of the new target.

The data acquisition started when the target (cross) jumped to the periphery and lasted 1 s. The sampling frequency was 300 Hz. Data from both eyes were recorded simultaneously. Each recorded data point had a time stamp and a validity code. After the data acquisition, the target jumped back to the center of the screen.

### Data Processing

The recorded data was processed offline. Trials in which more than 10% of the data was missing (validity code higher than 1 as provided by the eye tracker) or more than 100 ms was missing continuously or more than 80% was missing in the first 50 ms were excluded from further analysis. In the rest of the trials, missing values were interpolated with linear interpolation of the neighboring values.

The preprocessed data were smoothed and differentiated with a 0, 1^st^ and 2^nd^ order 11-points sliding window Savitzky-Golay filter^3^ to calculate the position, velocity and acceleration of the eyes. Saccades were detected automatically: if the velocity of the eye exceeded 50°/s in 2 consecutive points it was labeled as a saccade like event. The beginning of the saccade like event was marked where the acceleration of the eye was 0 (or reached its minimum value in 50 ms before its peak). The end of the saccade like event was marked where its velocity reached zero after the peak. Saccade like motion was accepted as a saccade if its latency occurred between 100 and 600 ms after stimulus onset, it took at least 12 ms and a fixation preceded the saccade like event. During fixation the eye had to be close to the initial cross (<1.5°) and its position change over the fixation had to be <0.6°. For all trials the position-time diagram was checked visually and inadequate trials were excluded. The first two trials in each condition were seen as practice and excluded from further analysis. A condition was accepted if the subject had at least 9 trials (half of the trials in a condition) after exclusion. Saccade latency (the start of the saccade), saccade peak velocity, saccade amplitude and saccade duration were assessed. Saccade gain was calculated from the ratio of the final eye position and the target position. Anti-saccade latency, gain, peak velocity were determined similarly in the correctly performed trials. Anti-saccade performance was calculated as the percentage of correctly performed trials to all the adequate trials. Moreover, a dysconjugacy index (DI) was calculated in the saccade task from both eyes as the ratio of the abducting and adducting eye's velocity in the “long” condition. DI was determined in the left and right directions. Patient's *Z*-scores were calculated as indicated in Equation 1.

(1) $$\begin{array}{l} Z_{DI}=\frac{DI\left(\text{MS}\right)-mean\left(DI\left(HC\right)\right)}{STD\left(DI\left(HC\right)\right)} \end{array}$$

### Magnetic Resonance Imaging

Magnetic resonance imaging was performed with a 3 T GE Discovery 750 w MR Scanner (GE Healthcare, Chalfont St. Giles, UK). The MR images used in the current study were acquired as part of the routine follow up of the patients, the protocol of which is described in details in our recent recommendation (12). The following sequences were used in the current analysis: High resolution T1 weighted anatomical images (3D spoiled gradient echo images with inversion recovery (3D FSPGR IR: echo time [TE]: 2 ms; repetition time [TR]: 5.4 ms; inversion time [TI]: 450 ms; matrix: 256 ^*^ 256; field of view [FOV]: 25.6 cm ^*^ 25.6 cm; flip angle: 12°; slice thickness [sl]: 1 mm; PURE intensity correction), CUBE T2 FLAIR for lesion detection (TE: 135 ms; TR: 6700 ms; TI: 1827 ms; matrix: 256^*^224; FOV: 25^*^22.5 cm, sl: 1.4 mm; fat sat; post processing: ZIP512, ZIP2), CUBE double inversion recovery (DIR) (TE: 90 ms; TR: 7,000 ms; TI: 2,901 ms; blood suppression TI: 546 ms; matrix: 192^*^192; FOV: 25 cm ^*^ 25 cm; sl: 1.4 mm; fat sat) and spin echo (SE) T1 weighted images were acquired (TE: min full, TR: 500, flip angle: 73°; matrix: 256^*^224, FOV: 24 cm ^*^ 19.2, sl: 3 mm, NEX: 2).

### Image Analysis

Lesion load was determined in the periventricular, infratentorial, and juxtacortical regions on the FLAIR and DIR images manually. Lesion load in the whole brain as well as in the above-mentioned subregions was correlated with the behavior parameters. The SE T1 images were used for determine black hole burden.

The correlation of lesion location probability and eye movement deficit was evaluated as described by Kincses et al. (13). Binary lesion mask were brought into standard space by registering the FLAIR images to the high resolution T1 weighted images by 6 DOF linear registration (14) and the T1 weighted images to standard MNI152 space by non-linear registration (15). The standard space binary lesion masks were concatenated. A voxelwise GLM analysis was performed, the regressors of the design matrix were the measured eye movement parameters. Non-parametric permutation test, with 5,000 permutations were used for statistical inference with correction for multiple comparisons.

The high resolution T1 weighted images were used for voxel-based morphometry analysis. We employed an “optimized” VBM-style protocol (16, 17) using FSL (18). Non-brain parts were removed from all structural images (19) and tissue-type segmentation was carried out by FAST4 (20). The resulting gray matter partial volume images were registered to standard space (MNI152) using linear transformation (14) followed by a non-linear registration (15). The resulting images were averaged to create a study-specific template, to which the native gray matter images were then non-linearly re-registered. The registered partial volume images were then modulated (to correct for local expansion or contraction) by dividing by the Jacobian of the warp field. The modulated segmented images were then smoothed with an isotropic Gaussian kernel with a sigma of 2 mm. Finally, voxelwise GLM was applied and permutation-based non-parametric testing correcting for multiple comparisons across space was used for statistical inference. The design matrix contained the behavior parameters (saccade latency, peak velocity, and gain) in consecutive analyses. The model was adjusted for disease duration and age. Thresholding was carried out by cluster-based thresholding corrected for multiple comparison by using cluster size.

### Statistical Analysis

Statistical analysis was carried out with Rstudio (21). The following packages were used: *lme4* (22)—model building, *car* (23)—statistical significance. Mean and standard error were calculated for the following parameters: latency, peak velocity and gain from both eyes in the prosaccade and anti-saccade tasks separately. All parameters were evaluated in a mixed model ANOVA, in which the subject was the random effect and the group (HC-MS), the movement type (abduction-adduction), and the distance (far-close) of the target handled as fixed effects. A *p* < 0.05 was considered significant. We investigated both eyes separately because an average of the two eyes could be misleading if subclinical internuclear ophthalmoparesis coexists. The results from the left eye were reported unless otherwise stated. To investigate oculomotor decision, latency and peak velocity differences between prosaccade/anti-saccade tasks were calculated and compared between the groups. The name of the new calculated variables were peak velocity difference and latency difference. As peak velocity and amplitude have linear relationship in case of significance the statistic was repeated with the scaled peak velocities. Pearson or Spearman correlation (where the assumptions of the Pearson were not valid) between MRI markers and behavior parameters were calculated in separate analyses. The effect of disease duration and age was tested in partial correlation and the effect of sex is tested via comparing the Fischer Z transformed correlation coefficients in the two sex separately. Correlation coefficient are reported from simple correlation where the age, disease duration and sex had no effect on the association.

## Results

Clinical examination indicated that 5 patients (13%) had clinically detectable oculomotor alteration. They were excluded from further quantitative analysis. One further patients was also excluded because of technical issues with the MRI images. The demographical data of subjects are presented in Table 1. All patients were on disease modifying therapy (6 patients take dimethyl-fumarate, 11-teriflonomide, 4- i.m. interferon beta1a, 7-glatiramer acetate, 10-fingolimod, 1-s.c interferon beta1a).

The average total lesion number was 21 (±15). As expected, most of the lesions occurred in the periventricular region (12.5±7.6), but significant lesion load was found in the infratentorial and juxtacortical location too (1.5 ±1.6, 3.6 ±4.5, respectively).

### Latency

The latency of the anti-saccades were longer than the latency of saccades (194 ms vs. 303 ms, *t* = −17.3, *p* < 0.0001 for HCs and 207 ms vs. 319 ms, *t* = -18.6, *p* < 0.0001 for MS participants).

**Figure 1:**
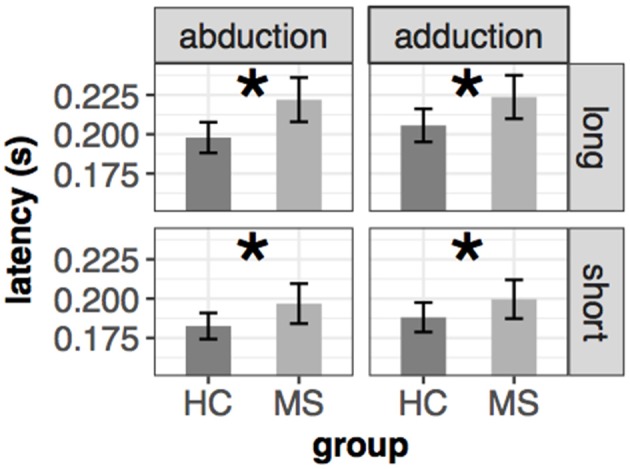
Mean and ±SEM of latency in different conditions of the left eye in the saccade task. Significant group difference is marked with an asterisk (*). The mean and standard error from the left-upper part of the figure were the following: 198(±5)ms vs. 221(±7)ms (abduction+long), 206(±5)ms vs. 224(±7)ms (adduction+long), 183(±4)ms vs. 197(±6)ms (abduction+short), 188(±5)ms vs. 200(±6)ms (adduction+short) for HC and MS, respectively.

Saccade latency was significantly prolonged in MS patients. The results were similar in both eyes [left: *F*_(1, 65.966)_ = 5.36, *p* = 0.024, right: *F*_(1, 65.98)_ = 5.38, *p* = 0.024] [mean(±sd): 194(±24) ms vs. 207(±31) ms for HC and MS participants]. There were no interaction effects between the fixed effects (group, movement type and distance of the target). The results are depicted in Figure 1.

Anti-saccade latency was prolonged in MS patients, however it did not reach a significant level [*F*_(1, 64.96)_ = 2.39, *p* = 0.12] [mean(±sd):303(±44) ms vs. 319(±44) ms for HC and MS, respectively].

The latency difference between prosaccade and anti-saccade tasks was also investigated on group level and we found no significant difference. [*t*_(64)_ = 0.29, *p* = 0.77] [mean(±sd): 107(±36) ms vs. 112(±34) ms for HC and MS, respectively].

### Peak Velocity

**Figure 2:**
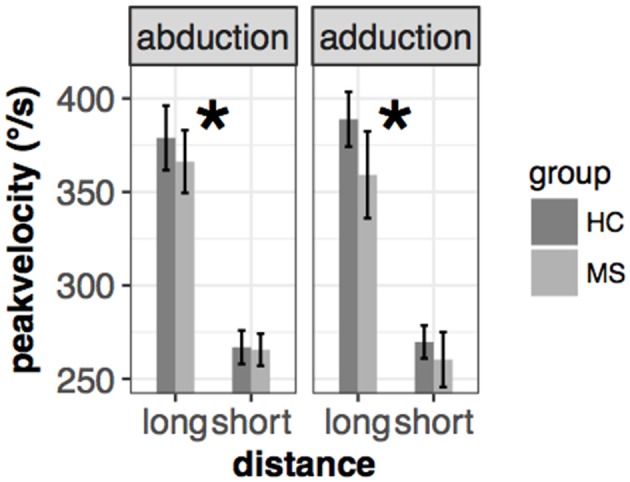
Mean and ± SEM of peak velocity in different conditions of the left eye in the saccade task. Significant group* distance interaction is marked with an asterisk (*). The mean and standard error are the following from left to right: 379(±9)/s vs. 366(±8)/s (abduction+long), 266(±4)/s vs. 265(±4)/s (abduction+short), 389(±7)/s vs. 359(±12)/s (adduction+long), 269(±4)/s vs. 260(±7)/s (adduction+short) for HC and MS, respectively.

Prosaccade peak velocity was slightly smaller in the MS group [mean(±sd): 325(±32)°/s vs. 310(±42)°/s for HC and MS, respectively] but there was no significant difference between the two groups [*F*_(1, 65.96)_ = 1.4, *p* = 0.28]. However, a significant interaction effect could be observed in two conditions: (i) group x movement type [left eye: *F*_(1, 187.1)_ = 3.3, *p* = 0.071, right eye: *F*_(1, 192.7)_ = 7.2, *p* = 0.0079], which means that the higher velocity of adduction compared to abduction in HC was reversed in MS patients, resulted in a higher peak velocity in abduction compared to adduction and (ii) group x distance pleft eye: *F*_(1, 187.1)_ = 7.7, *p* = 0.006, right eye: *F*_(1, 192.2)_ = 7.15, *p* = 0.008], which means that the slower peak velocity in the closer cue condition in HC group was slightly smaller in the MS group (Figure 2).

Anti-saccade peak velocity was not different between the two groups [*F*_(1, 64.8)_ = 0.12, *p* = 0.73] [mean(±sd): 265(±46)°/s vs. 268(±41)°/s for HC and MS, respectively]and no interaction effects could be observed.

Difference between prosaccade and anti-saccade peak velocity could be observed in a group level. MS group had lower difference in peak velocity around 20/s [*t*_(64)_ = 2.13, *p* = 0.037] [mean(±sd): 60(±33)°/s vs. 42(±37)°/s for HC and MS, respectively], however, this difference was not survived scaling for amplitude [*t*_(64)_ = 0.07, *p* = 0.95].

Moreover, EDSS scores positively correlated with peak velocity difference (Spearman's rho: 0.4, *p* = 0.024). The higher clinical disability related to higher peak velocity difference between the two tasks.

### Gain

**Figure 3:**
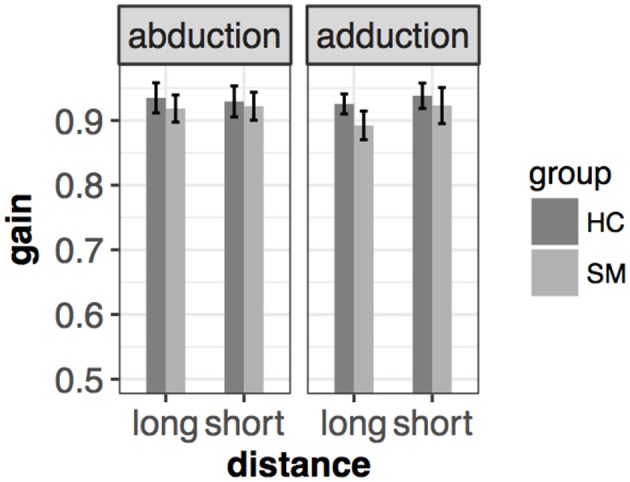
Mean and ± SEM of gain in different conditions of the left eye in the saccade task. Slight hypometria could be detected, however, this difference was not significant. Mean and standard error are the following from left to right: 0.935(±0.012) vs. 0.918(±0.11) (abduction+long), 0.929(±0.012) vs. 0.922(±0.011) (abduction+short), 0.926(±0.008) vs. 0.89(±0.011) (adduction+long), 0.938(±0.01) vs. (adduction+short) for HC and MS, respectively.

In the prosaccade task, MS group had smaller gain in all conditions. Therefore, they performed slightly hypometric saccades compared to HC. However, this difference did not reach a significant level [*F*_(1, 65.8)_ = 2.24, *p* = 0.14] [mean(±sd): 0.932(±0.046) vs. 0.913(±0.049) for HC and MS, respectively] (Figure 3).

Gain in the anti-saccade task did not differ between the two groups [*F*_(1, 63.6)_ = 0.01, *p* = 0.92].

### Antisaccade Performance

**Figure 4:**
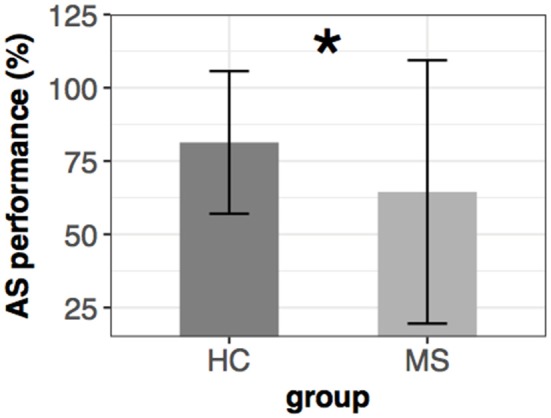
Mean ± SEM of anti-saccade performance in the two groups. Significant group difference is marked with an asterisk (*).

There was a marked difference between the two groups in the anti-saccade performance. The HC group reached more than 80% (±12.2%) accuracy while the MS group obtained only 64% (±22.5%). (Wilcoxon-Mann-Whitney test: [*U*_34, 33_ = 837, *p* < 0.001] (Figure 4).

### Dyconjugacy Index

**Figure 5:**
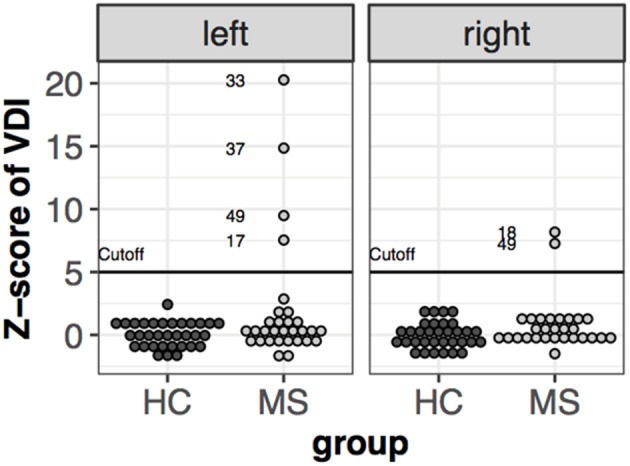
*Z*-scores of velocity dysconjugacy index individually, Subjects' value higher than the cut-off are labeled.

The dysconjugacy index derived from peak velocities of the eyes can detect clinically not detectable internuclear ophthalmoparesis (INO). A *Z*-value higher than the highest control subject's *z*-value+2 was determined as threshold for subclinical INO (10). Based on this threshold five patients were classified as having subclinical INO. Patient #49 had INO in both directions, while patient #39, #37, and #17 had only in the left direction and patient #18 only in the right direction (Figure 5).

### Correlation of Eye Movement Deficit With MRI Markers

T2 lesion burden or lesion location did not show significant correlation with any of the measured MRI parameters.

**Figure 6:**
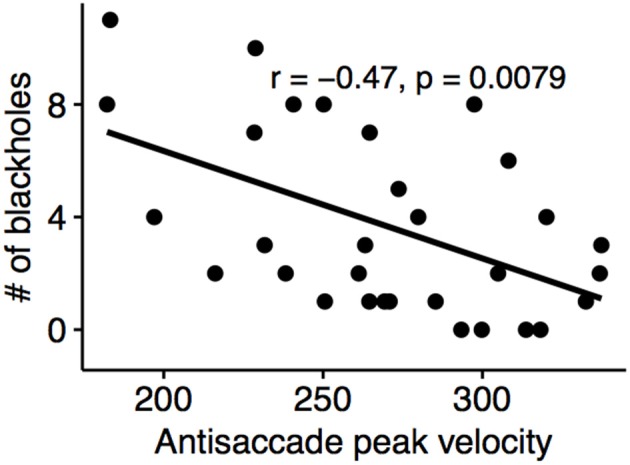
Antisaccade peak velocity of the left eye negatively correlated with black-hole count.

A positive correlation were detected between anti-saccade latency and the number of black-holes (Spearman's rho: 0.45, *p* = 0.011) and negative correlation between the anti-saccade peak velocity and the number of black-holes (Spearman's rho: −0.47, *p* < 0.01) (Figure 6).

There were no significant correlation between white matter volume with any of the measured eye movement parameters.

**Figure 7:**
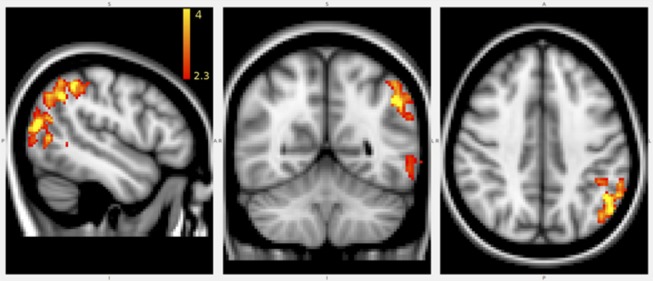
Results of VBM analysis. Marked voxels positively correlated with anti-saccade peak velocity. The error bar represents different *Z*-value after cluster based thresholding. Disease duration and age were used as cofounders.

The VBM analysis revealed that anti-saccade peak velocity correlated with gray matter density in parietal areas (Figure 7). That is, smaller anti-saccade peak velocity was associated with lower gray matter densities in the left parietal areas.

No other eye movement parameters showed correlation with gray matter density.

## Discussion

In our study we investigated visually guided prosaccade and anti-saccade task performance in MS patients and their possible association with focal brain alterations. Out of several eye movement parameters, we found significantly increased latency in the prosaccade task and significantly worse performance in the anti-saccade task in MS patients. The detailed examination of conjugated eye movements revealed 5 subclinical INO cases.

As regarding the MRI parameters, the peak velocity and latency of the anti-saccade movement correlated with the number of black-holes, but none of the eye movement parameters were associated with the T2 lesion burden or location. Most importantly, local gray matter atrophy in the left inferio-parietal lobule and temporo-parietal junction correlated with anti-saccade peak velocity.

Oculomotor alterations found with various paradigms such as visually guided saccade, prosaccade, anti-saccade, memory guided saccade and endogenous cued saccade are common in MS (1, 3, 10, 24–31). In agreement with our findings, Clough et al. found that saccade latency is prolonged in clinically definitive MS patients (24). In the same cohort, latency increases with longer disease duration. In another study, the MS group has longer saccade latency in the presence of a distractor stimulus (29). Previous studies also showed that the performance is deteriorated mainly in more cognitively demanding saccade tasks (3, 24–28, 31). Antisaccade performance is deteriorated and associated with cognitive performance (24, 28, 31). Fielding claims that this alteration spares the reflexive part of the saccades. In our investigation the anti-saccade peak velocity, but not that of the parameters of the saccade task correlated with number of black-holes and focal gray matter atrophy in the temporo-parietal region. Prolonged latency of prosaccade could mirror the delayed initiation of saccades. The prolonged latency of anti-saccades however, might reflect a prolonged volitional decision process or a delayed initiation of saccade in the opposite direction or both (32). The difference might relates to the time, which is not necessary for the reflexive part such as inhibition or vector transformation (33). Hence the correlations we have found are mainly reflecting the higher order cognitive processes of eye movements rather the reflexive parts. Interestingly, no correlation was found between any of the MRI parameters and the anti-saccade performance. The non-reflexive part of the anti-saccades might be dysfunctional, leading to an error. While if it is delayed but to a level that is not sufficient to make an error it could only be investigated via its delayed latency. This could especially be important in multiple sclerosis, in which demyelination, and slowed conduction is a key feature of the disease.

Saccades could be a potential marker to follow-up cognitive alterations in MS patients because it has been shown that various saccade performances are associated with cognition (24, 25, 27). Based on these observations, prolonged latency could reflect damage to networks associated with motor or cognitive control.

While several studies showed alterations of oculomotor performance in MS, the background of such alterations is not entirely clear. Clinically detectable oculomotor symptoms show correlation with certain infratentorial lesions (9), but in our investigation, subclinical eye movement deficit did not correlate with T2 hyperintense lesion load, which is congruent with the result of a previous study (31). It is in agreement with our earlier investigation in that lesion load or location only modestly correlate with clinical symptoms (13), whereas persistent black-holes show better correlation with clinical and cognitive functioning in MS (34, 35). Accordingly, the T1 hypointense lesion burden correlated with the anti-saccade velocity and latency in our study. Brain atrophy showed better correlation with clinical and cognitive disability (36). In particular, cerebellar atrophy was associated with anti-saccade error (31). In our study, gray matter atrophy measures correlated with eye movement deficits globally as well as locally. Black holes and atrophy seem to jointly relate to anti-saccade alterations. Several studies found correlation between T1 black-hole lesions and atrophy, but no similar relationship was revealed for T2 hyperintense lesions (37, 38). Cellular damage could be observed in both cases (39, 40). In addition, both measures correlate well with clinical disability, better than T2 lesion load (41).

Saccade peak velocity is affected by multiple cognitive functions [arousal (42) and mental workload (43)]. In our study, focal gray matter volume variability showed correlation with anti-saccade peak velocity in the left inferior parietal lobule, left temporo-parietal junction and in the putative left V5/MT motion sensitive visual region (44). These parietal regions are identical to those frequently implicated in attention tasks (45). In their seminal paper, Corbetta et al. in a remarkably similar paradigm found activation in the intraparietal sulcus during sustained attention and in the right temporo-parietal junction when a target was detected, particularly at an unattended location (4). These two conditions correspond to the top-down and bottom-up attentional subsystems. Moreover, the parietal cortex has its direct connection to the superior colliculus (6–8), and the pontine nuclei as well (46). Damage to these perisylvian regions was also implicated in neglect (47). Visuo-spatial neglect in MS patients has been described, but no associated structural damage has been found so far (48). Interestingly, the right temporo-parietal junction is implicated in target detection, but in our study the atrophy of the left temporo-parietal junction was associated.

Alternatively, parietal region has a potential role in saccades. The parietal eye field and posterior parietal cortex are involved in saccade generation (49) and visuospatial attention (45). Moreover, human (50) and animal (51) studies suggested that this region has a role in the vector inversion process, which is a crucial step in anti-saccades.

In conclusion, saccades are substantially affected in MS patients, which reflected in several behavior parameters. Global and focal gray matter alterations are associated with brain areas important in cognitive functions, such as attention.

## Ethics Statement

This study was carried out in accordance with the recommendations of the Medical Research Council National Scientific and Ethical Committee (ETT TUKEB) with written informed consent from all subjects. All subjects gave written informed consent in accordance with the Declaration of Helsinki. The protocol was approved by the National Institute of Pharmacy and Nutrition (000002/2016/OTIG).

## Author Contributions

BK, NS, and ZK: designed the study. BK, BH, BB, KK, PF, AK, DV, ET, and NS: collected and organized the database. ZK, KB, and LV: recruitment and interpretation of the data. BK and BH: performed the statistical analysis. BK and ZK: wrote the manuscript. All authors contributed to manuscript revision, read and approved the submitted version.

### Conflict of Interest Statement

The authors declare that the research was conducted in the absence of any commercial or financial relationships that could be construed as a potential conflict of interest.

## Footnotes

^1^http://developer.tobiipro.com/matlab.html

^2^http://psychtoolbox.org/

^3^https://www.mathworks.com/matlabcentral/fileexchange/30299-savitzky-golay-smooth-differentiation-filters-and-filterapplication
